# Supplementary material for: Antenatal screening of depressive and manic symptoms in south Brazilian childbearing women: A transversal study in advance of the pandemic scenario
Source: PLoS One. 2021 Dec 28;16(12):e0261874. doi: 10.1371/journal.pone.0261874 (PMC8714122; doi:10.1371/journal.pone.0261874)
Supplement: Attachment — Submitted filename: Response to reviewers2.docx [file pone.0261874.s002.docx]

**Response to the reviewers**

Dear Editor,

Raoul Belzeaux

Plos One

Thank you for the pertinent review and for the opportunity of improving our paper entitled “Antenatal screening of depressive and manic symptoms in south Brazilian childbearing women: A transversal study in advance of the pandemic scenario.”

Please find below our answers. The changes are highlighted in yellow in the manuscript.

**Reviewer#1**

**Remark1#** Table 3, symbol n(%) to be added. For total, 100% to be added apart from total number.

**Our answer:** The suggestion has been included in Table 3. Thank you.

**Remark 2#** Line 264, typo 'countries. [29-32] Therefore''

**Our answer:** It has been corrected. Thank you.

**Reviewer #2:** I have no futher comment about this study. All my comments about the previous round review were corrected.

**Our answer:** Thank you.

Thank you again for the useful suggestions. We are willing to elucidate and improve any doubts or suggestions that may persist.

Regards,

Fernanda Schier de Fraga and Team.
